# Platelets: Still a Therapeutical Target for Haemostatic Disorders

**DOI:** 10.3390/ijms151017901

**Published:** 2014-10-07

**Authors:** Reinaldo Barros Geraldo, Plínio Cunha Sathler, André Luiz Lourenço, Max Seidy Saito, Lucio M. Cabral, Pabulo Henrique Rampelotto, Helena Carla Castro

**Affiliations:** 1 Programa de Pós-graduação em Ciências e Biotecnologia, Instituto de Biologia, Universidade Federal Fluminense (UFF), Niterói CEP 24210-130, RJ, Brazil; E-Mail: reinaldobgeraldo@yahoo.com.br; 2 Programa de Pós-graduação em Patologia, Departamento de Patologia, Hospital Universitário Antônio Pedro (HUAP), Universidade Federal Fluminense (UFF), Niterói CEP 24030-215, RJ, Brazil; E-Mails: pliniocs@yahoo.com.br (P.C.S.); andrebiouff@gmail.com (A.L.L.); maxs@yahoo.com.br (M.S.S.); 3 LabTIF, Faculdade de Farmácia, Universidade Federal do Rio de Janeiro (UFRJ), Rio de Janeiro CEP 21941-590, RJ, Brazil; E-Mail: pliniocs@yahoo.com.br; 4 Interdisciplinary Center for Biotechnology Research, Federal University of Pampa, Antônio Trilha Avenue, P.O. Box 1847, São Gabriel/RS 97300-000, Brazil

**Keywords:** platelets, agonists, receptor, thrombosis, aspirin^®^

## Abstract

Platelets are cytoplasmatic fragments from bone marrow megakaryocytes present in blood. In this work, we review the basis of platelet mechanisms, their participation in syndromes and in arterial thrombosis, and their potential as a target for designing new antithrombotic agents. The option of new biotechnological sources is also explored.

## 1. Introduction

Platelets are enucleated subcellular fragments from megakaryocytes of bone marrow [1,2,3]. In the human body, nearly 70% of the platelets are present in the blood circulation and 30% are stored in the spleen. They are the smallest components of the circulating blood (2–3) µm in a concentration of (150–400) × 10^9^/L and have a life span of 7–10 days [3,4,5], when they are removed by reticuloendothelial cells from the spleen and liver [6,7].

Platelets present an important role in the haemostatic system and in several pathologies (e.g., cerebral ischemia and arterial thrombosis) [8,9,10,11]. These pathologies are related to platelets’ unspecific activation, promoting agonists secretion and platelet aggregation in atheromas plaques, leading to a decreased circulation and an ischemic region. In the present study, we review the physiological basis for platelets, including platelet structure and function. In addition, we also highlight the importance of platelets in some diseases and their use as a therapeutic target for designing new antithrombotic agents.

## 2. Platelets–Complex Structure

Despite their vesicle appearance in the peripheral blood, platelets are subcellular fragments with cytoplasmatic and granular content plus complex and organized structure. Structurally, platelets may be divided into four zones based on organization and function: (1) Peripheral zone; (2) Sol-gel zone; (3) Organelles zone; and (4) Membrane zone or system [12,13].

**Figure 1:**
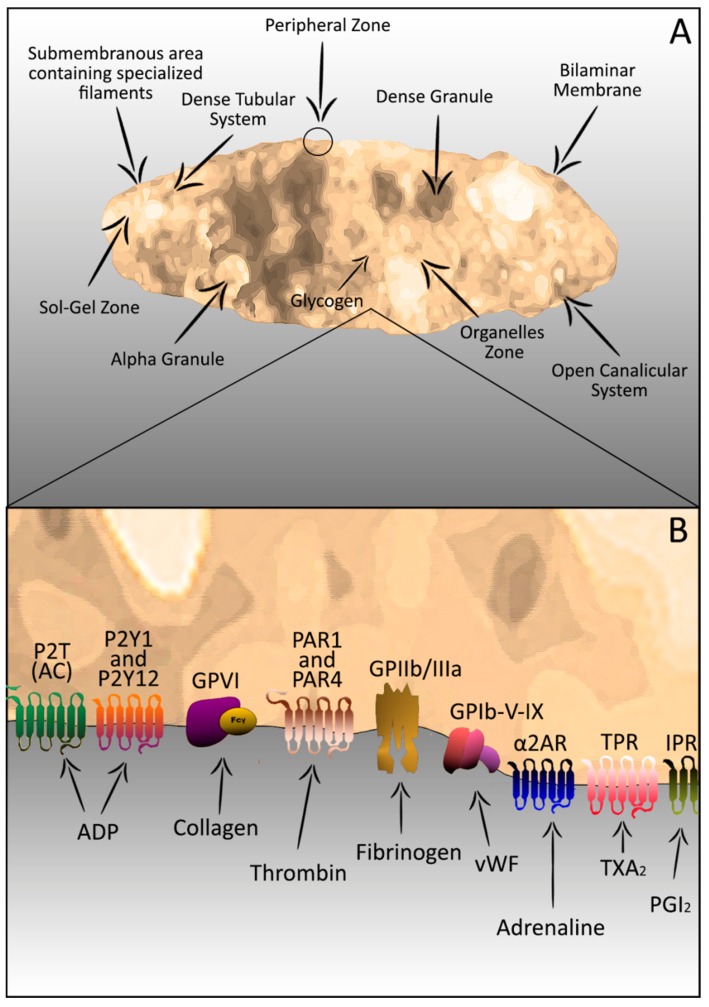
Platelets: Components and organization (**A**); and receptors and agonists (**B**).

(1) The peripheral zone consists of the extra and intracellular cell membrane covered by a thick surface coat of glycocalyx and a canalicular system linked to the surface called open canalicular system (OCS). The OCS is responsible for the molecular exchanging with extracellular environment, where occurs a significant release of molecules during the platelet secretion process after activation [14,15]. Despite the characteristic changes in the membrane release region, the release of granular contents occurs without platelet rupture and with the maintenance of the membrane integrity [14,16]. The platelet membrane is an asymmetrical phospholipids bilayer (inner and outer leaflet) where phosphatidylcholine is highly distributed; sphingomyelin is found exclusively in the outer leaflet while phosphatidylethanolamine, phosphatidylcholine, and phosphatidyinositol are present in the inner leaflet. This membrane is rich in glycoproteins (GP), which are targets for adhesion reactions or receptors for initiating the platelet activation [17,18]. Furthermore, membrane phospholipids can be found, acting as a surface for some coagulation factors. Some of these phospholipids are used as substrates for arachidonic acid production and thromboxane A_2_ production (TXA_2_). These compounds are potent agonists in platelet aggregation and vasoconstriction processes [11,17].

(2) The sol-gel zone is located beneath the peripheral zone and is composed of an actin cytoskeleton (responsible for platelet shape change after activation) and microtubules (necessary to maintain the discoid shape) [19,20]. Initially, the phosphorylation of regulatory myosin light chains (MLC) on serine 19 of myosin IIA stimulates the ATPase activity that allows the interaction of myosin with actin filaments. Interestingly, platelet agonists stimulate the coordinated signaling process responsible for the simultaneous activation of two regulatory enzymes: MLC kinase (MLCK) and MLC phosphatase. These enzymes activate the phosphorylation of MLC. Then, microtubules direct the granular centralization for the release of their contents through the OCS. This event differs from classic cells exocytose where fusion occurs directly between the granular contents and the plasmatic membrane [12,21]. Finally, the actin–myosin interaction leads to contraction of the actin cytoskeleton required for shape change, pseudopodia extension and granular secretion [19,20,22] (Figure 1).

(3) The organelles’ zone occupies the center of the platelet. It is composed of α granules, dense granules and cellular contents. These granules are responsible for 30%–50% of all proteins present in the platelet. The α granules contain adhesive proteins, von Willebrand factor (vWF), thrombospondin, vitronectin, platelet-derived growth factor (PDGF), platelet factor IV, coagulation factors (e.g., Factor XI and XIII) [3,23,24], and plasminogen activation inhibitor (PAI). The dense granules are rich in adenosine triphosphate (ATP), adenosine diphosphate (ADP), serotonin, and calcium ions. Finally, the cellular contents include lysosomes and mitochondria [13], which contain ATP and ADP, store enzymes, and are responsible for other molecules relevant to the platelet functions.

(4) The membrane zone or system includes a dense tubular system that stores calcium ions (Ca^++^) vital for contractile events and an enzymatic system involved in prostaglandin synthesis production [25,26].

## 3. Platelets in the Haemostatic Process

**Figure 2:**
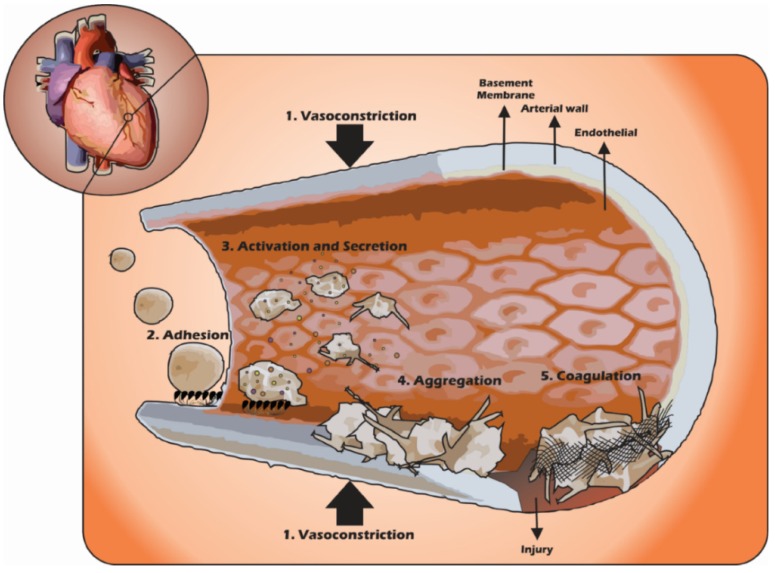
Participation of platelets during the formation of platelet plug in haemostasis. 1: Vasoconstriction; 2: Platelet adhesion to subendothelium; 3: Platelet shape change with secretion granules and 4: Binding platelet/platelet; and 5: Fibrin deposition on the platelet plug.

The haemostatic system is responsible for blood circulation maintenance and vascular integrity. This system is able to form a plug on an injured surface in the vascular endothelium, which minimizes blood loss and recovers the vascular structure [27,28]. This plug is a multi-cellular process product that involves platelets and other blood cells, such as leukocytes and endothelial cells (Figure 2).

Platelets, coagulation factors, fibrinolytic factors, protein inhibitors and endothelial cells constitute the haemostatic system, each one with specific roles for the haemostatic activity. Platelets are responsible for the formation of a platelet aggregate [3,5,24,29], which initiates the haemostatic plug. The coagulation factors group is composed of pro-enzymes produced by the liver that are involved in the coagulation cascade process. They are activated during the haemostatic process, leading to the fibrin polymer formation, “covering” the platelet plug. The fibrin polymers and the coagulation factors are controlled by the fibrinolytic factors (e.g., plasmin) and protein inhibitors (e.g., anti-thrombin), responsible for the extension of the coagulation plug, preventing abnormal spreading.

The endothelial cells lining the blood vessels contribute to the maintenance of blood circulation. The avoidance of thrombus formation is somehow connected with the similar charge properties of platelets and endothelial cells, which generates repulsion between them. In addition, the endothelial cells synthesize nitric oxide (NO) and prostacyclin, important platelet inhibitors, thrombomodulin, which inhibits thrombin and heparin sulfate that activates antithrombin III [24,30].

In the physiological state, platelets circulate without adhering to undisturbed vascular endothelium. In case of the vascular endothelial integrity disruption (e.g., shear stress of the blood flow), platelets are “activated”. During vascular endothelial rupture, some proteins are exposed in the subendothelium (e.g., Collagen, vWF, fibronectin and laminin) at the site of injury. Then, platelets bind to collagen and vWf, mediated by collagen receptors (α_2_β_1_ integrin and GPVI) and vWF receptor (GPIb) [3,24,31]. In high shear conditions (arterial shear rates), platelet GPIb and vWF interaction induces platelets’ conformational change (shape change) and the activation of platelet integrins α_IIb_β_3_ and α_2_β_1_. Then, these integrins bind to collagen leading to attachment, adhesion and platelet aggregation [32,33,34]. Differently, in low shear conditions (venous shear rates), platelet adhesion does not depend on GPIb, and integrin α_IIb_β_3_ plays the main role. On resting platelets, α_IIb_β_3_ is in an inactive state, but activation of platelets by signaling from GPIb-V-IX or GPVI/collagen causes “inside-out” activation of α_IIb_β_3_ [32,34,35]. The activated platelets release several agents that recruit additional platelets to the injury site, leading to the consolidation of the haemostatic plug aggregate [36]. This activation process initiates through a range of specific cell surface receptors associated to intracellular signaling pathways.

Collagen and vWF may be considered as primary haemostatic agonists, whereas thrombin (generated by the coagulation cascade), ADP (released from platelet dense granules), and TXA2 (synthesized and released by activated platelets) are the secondary agonists [37,38,39,40].

The recruitment of additional platelets occurs through platelet–platelet interaction that is mainly mediated through fibrinogen and α_IIb_β3 receptor.

Laboratory tests have been used to identify and classify disturbances in haemostasis and the platelet function [3,41,42,43,44]. Usually the tests initiated with analysis of the peripheral blood, checking the morphology and the number of platelets, white and red blood cells. Other tests include platelet aggregation (light transmission aggregometry—LTA), bleeding time (BT) and secretion assays, using the whole blood or platelet-rich plasma (PRP). Moreover, new platelet function tests such as laser platelet aggregometer (PA-200), platelet function analyser (PFA-100) and rapid platelet function analyser (RPFA—Ultegra) show potential both in research or as point of care instruments (defined as a test requiring only one single pippeting step or as bedside tests that can be performed by non-laboratory personnel [45]).

For evaluating *in vitro* platelet aggregation, the gold standard is the light transmission aggregometry (LTA). It still remains as the most used test for the identification and diagnosis of platelet function problems, allowing a more precise characterization [20,46]. In this procedure, PRP is stirred within a cuvette located between a light source and a detector. Then, the agonist (ADP, epinephrine, collagen, arachidonic acid, ristocetin or thrombin) is added, leading to platelet aggregation and increased light transmission (Figure 4). Parameters measured include the slope of aggregation (%/min) and the maximal amplitude (%) after a fixed time [20,46].

**Figure 3:**
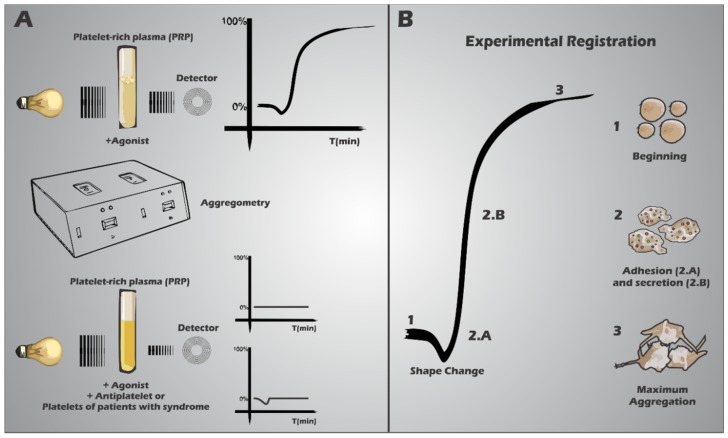
Tests of platelet function through light transmission aggregometry (**A**) and its experimental record (**B**). (**A**) Normal platelets (up), disabled or treated with the antagonists (down) are activated by agonists such as ADP, thrombin and arachidonic acid, resulting in different records; (**B**) experimental graphical profile of aggregometry assay using normal platelets, appearing at (1) the beginning of the test after adding the agonist, followed by (2.A) shape change. Then, platelets’ adhesion and aggregation occur (2.A—primary aggregation) and if the stimulus is adequate, there is the granule secretion (2.B—secondary aggregation) and the maximum platelet aggregation (3).

The aggregation profile initially displays the platelet shape change (from discoid to spherical form) increasing light scattering through the PRP. Then, the primary aggregation occurs, characterized by the platelets adhesion and aggregation, which decreases light scattering and increases light transmission [20,46]. Finally, as the stimulus continues, platelets release their granule contents. The graph trace will continue to increase, representing the maximum aggregation (secondary aggregation) with maximum light transmission [20,46] (Figure 3).

The pattern obtained usually can diagnose and identify the aggregation deficient profile [42,43,44]. A new version of the recently produced aggregometer is Platelet works (Helena, Beaumont, TX, USA) designed to determine the platelet aggregation level (platelet number and activity) during cardiac surgery procedures [42].

The BT result is operator-dependent and is affected by age and skin texture of the patient. Both BT and PFA-100 time are prolonged in patients with low hematocrit and normal platelet function. Despite the limitations of BT and PFA-100 assays, these tests can be useful for diagnosing patients with mucocutaneous bleeding.

The PFA-100 (PFA-100, Dade Behring, Marburg, Germany) and Ultegra (Accumetrics, San Diego, CA, USA) have also been used for the platelet function determination, since it eliminates the factors that interfere with aggregation observed in conventional assays and identifies patients with resistance to aspirin^®^, clopidogrel and inhibitors of αIIbβ3.

The platelet function analyzer PFA-100 is an equipment that checks the *in vitro* platelet function under high flow (5,000–6,000 s^−1^), simulating the conditions of arterioles. This is a model for simple and rapid assessment of platelet function, which uses cartridges that contain two agonists (collagen/ADP or collagen/epinephrine) [47,48]. It presents sensitivity to platelet count, haematocrit, drug effects, dietary effects, major platelet receptor defects, release defects and granular defects [47], specificity and reproducibility.

According to the literature, approximately 3%–5% of surgery patients have platelet defects and PFA-100 was capable of detecting impaired haemostasis in almost every case [47,49,50]. Therefore, it shows potential as a preoperative test and monitoring preoperative correction with pro-hemostatic agents [47,49,50], reducing the number of unnecessary blood transfusions [47,51]. Interestingly, the PFA-100 can be used to monitor the effectiveness of platelet transfusion therapy [47,52], but several studies showed problems in post-transfusion evaluation [53].

Differently, the Ultegra is a turbidimetry method using whole blood and polystyrene microparticles containing fibrinogen to allow binding of activated platelets. Its use is specific for the verification of the effects of the antagonists of GPIIb/IIIa (e.g., Abciximab, tirofiban or eptifibatide) and for monitoring patients in the intensive care units.

Other more expensive techniques may also be used for the detection of platelet disorders, including flow cytometry, electron microscopy and immunoelectrophoresis [42].

Abnormalities in the number or composition of platelets can initiate an imbalance in the early stages of the haemostatic system, resulting in bleeding tendency and impairment of platelet function [54,55]. Among the syndromes and diseases related to platelets are those involving receptor defects, granular disorders, deficient secretion, abnormalities in plasma factors that affect the platelet function and/or the interaction with platelet clotting factors.

Transfused platelets (pooled random-donor platelets or single-donor aphaeresis platelets) when stored for 5 days are equally efficacious and cost effective in reducing platelet transfusion requirements, transfusion-transmitted infections and bleeding disorders [56]. The major concern about this procedure is platelet alloimmunization, occurring when A and B red cell antigens are expressed in platelets. Another problem is red blood cells (RBC) alloimmunization, characterized by development of RBC antibodies such as anti-D, anti-C and anti-E [57].

There are several strategies for avoiding these complications, such as gamma irradiation, the standard care to avoid transfusion-associated graft-v*ersus*-host disease [58]. Newer strategies such as producing immortalized megakaryocyte progenitor cell lines from human pluripotent stem cells [59] are in line to overcome this problem. Despite that, efforts to minimize the plasma presence in platelet transfusion, to produce platelets analogues and to increase the effectiveness of platelet transfusions are still in need.

## 4. Platelet Receptor Defects

*Glanzmann Thromboasthenia* is autosomal recessive disease with a family bleeding history that is usually negative [60,61], characterized by the absence or decrease of receptor α_IIb_β_3_ expression [62,63,64], but with no change in number, size, shape or half-life of platelets. The treatment used is the transfusion of platelets, but the platelet alloimmunization is a serious “side-effect”.

*Bernard-Soulier syndrome* is an autosomal recessive disease characterized by larger platelets in small numbers, prolonged bleeding time and abnormal ristocetin aggregation due to the decrease or absence of receptors for vWF (GPIb-IX) [64]. Treatment with platelet transfusion is used therapeutically, however, similar to Glanzmann thromboasthenia, alloimmunization may occur [65,66].

## 5. Granular Disorders

Granular disorders are a heterogeneous group of diseases in which there is an abnormality in platelet capacity in store molecules within the granules [14]. These diseases can be associated with systemic disorders. Among the storage disorders associated with systemic disorders are: 

*Hermansky-Pudlak Syndrome—*Rare autosomal recessive disorder associated with oculocutaneous albinism [67]. It is characterized by bleeding for long periods. The dense granules show abnormalities and absence of ADP in platelets of metabolic origin. Studies of platelet functions showed aggregation on collagen deficient [68,69,70,71].

*Chediak-Higashi Syndrome*—Rare autosomal recessive disorder characterized by abnormal and large granules. It is similar to those found in melanocytes, leukocytes and fibroblasts. This deficiency shows a smaller number of dense granules and reduced aggregation associated with an abnormal tendency to bleed [72].

*Wiskott-Aldrich Syndrome*—Rare recessive disorder related to the Xp11.22-23 chromosome, characterized by thrombocytopenia with small platelets [73] and prolonged bleeding. Affected patients have a history of recurrent infections and eczema on physical examination. The treatment of acute bleeding consists of platelet transfusion and bone marrow transplantation as a final treatment for these patients [60,74,75].

## 6. Non-Storage Disorders Associated With Systemic Disorders

*Grey platelet syndrome* is characterized by deficiency of proteins in α granules in platelets and megakaryocytes. Among these proteins are the platelet factor IV, β-thromboglobulin, fibrinogen and PDGF. In the peripheral blood analysis, the platelets are large and gray [76].

*Quebec platelet disorder* is an autosomal dominant disorder associated with deficiency of aggregation induced by epinephrine. In particular, platelets have defects in the α granules proteolysis and multimerin, a multimeric protein that binds factor V and leads to smaller content of platelet factor V and other proteins such as fibrinogen, and vWF [76].

## 7. Secretion

This is the deficiencies’ largest group involving platelet functions. They are heterogeneous disorders caused by abnormalities in: (a) Membrane signal transduction; (b) Metabolic pathways; and (c) Mechanisms of secretion or in structures directly involved in the secretion of granular content after platelet activation [14]. These deficiencies are associated with prolonged bleeding time, and an abnormal profile *in vitro* in aggregation induced by ADP, epinephrine and collagen [76].

## 8. Arterial Thrombosis x Treatment

Besides hereditary disorders, platelets also play an important role in atherogenesis, ischemia, coronary artery thrombosis and other cardiovascular diseases, such as syncope, peripheral vascular disease and others related to mellitus diabetes. All these diseases involve vascular occlusion and direct participation of platelets [77,78,79,80,81,82,83,84,85].

Acute arterial occlusion is the blockage of blood circulation within a terminal artery, compromising the cellular metabolism in the affected areas. The ischemic situation may be severe, depending not only on the occluded artery, but also on the extent of ischemia, time and evolution of the clinical situation as well as the presence of a substitute collateral circulation. The affected area will determine the risk of the patient's life. Due to the death risk, the cause determination and the unclotting of the vessel should be made as early as possible to reverse the situation [86,87].

Venous thrombosis is characterized by red blood cells and a large amount of fibrin (red thrombus). This thrombotic process is generally initiated by the activation of the coagulation cascade. In contrast, the arterial thrombus adhered to sclerotic lesions is rich in platelets (white thrombus). The red thrombus is traditionally treated with anticoagulants (e.g., Heparin and warfarin) because of the direct relationship with the coagulation. For white thrombus, platelet inhibition has been the target for the treatment of acute coronary syndrome [81,88,89,90,91].

Currently, many drugs have been used as platelet antiaggregant agents in the treatment of arterial thrombosis. The most commonly used drug is the acetylsalicylic acid (aspirin^®^) [92,93,94,95,96], but other oral agents such as ticlopidine, clopidogrel, or dipyridamole, as well as intravenous platelet antiaggregant drugs such as abciximab or eptifibatibe, while the patient is under angioplasty procedure are still prescribed [97,98,99,100,101]. Each agent affects the platelets differently, presenting unique collateral effects. However, all cause reduction in both platelet adhesion and thrombus formation [43,87]. Platelet antiaggregant drugs differ from anticoagulants that act specifically on the factors of coagulation and their production. Therefore, if the patient is using an anticoagulant, the substitution by a platelet antiaggregant drug such as aspirin^®^ is not indicated [43].

**Figure 4:**
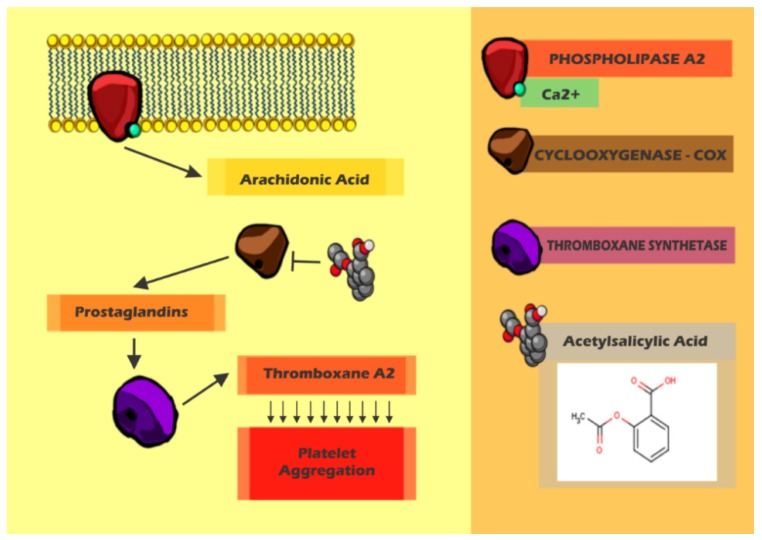
The Acetylsalicylic acid (aspirin^®^) and its mechanism. Production of arachidonic acid and thromboxane A_2_ pathway target for aspirin^®^.

Aspirin^®^ inhibits platelet aggregation, acting in a preventive manner in cardiovascular thrombotic events, becoming the most widely used cardiovascular drug due to both risk and cost. Aspirin^®^ and other non-steroidal anti-inflammatory drugs (NSAIDs) inhibit the metabolism of arachidonic acid through the inactivation of the cyclooxygenase enzyme (COX) [43,95,101] (Figure 4).

The arachidonic acid generated from the phospholipids membrane is converted into prostaglandin (PGG_2_) by platelet cyclooxygenase (COX-1) Figure 4). PGG_2_ is catalyzed by thromboxane synthase, leading to formation of TXA2 that promotes vasoconstriction and platelet aggregation (Figure 4). Aspirin^®^ acts by irreversible acetylation of the residue of serine at position 530 within the hydrophobic channel, which block the access of arachidonic acid to the catalytic site for platelet life spawn [95,102] (Figure 4). Other NSAIDs with low selectivity are reversible, acting as competitive inhibitors of the COX catalytic site [95,103].

The use of aspirin^®^ for primary prevention in low risk patients remains controversial because of the risk of bleeding and gastrointestinal bleeding episodes [104,105,106,107]. However, in cases of patients with the estimated risk of a cardiovascular event higher than 1% per year, the therapy with aspirin^®^ for cardiovascular protection is highly indicated [43,103,108] (Figure 4).

Thus, the use of aspirin^®^ has become routine in clinical practice in arterial thrombosis (cerebral and cardiac arterial disease), associated with drugs to prevent irritation of the gastric mucosa (e.g., ranitidine).

Thienopyridines development has significantly improved clinical outcomes in acute coronary syndrome (ACS) and in those undergoing percutaneous coronary intervention (PCI). The mechanism of action is related to selective and irreversible inhibition of P2Y_12_ purinoreceptor. This receptor is involved in platelet aggregation induced by ADP. The binding of ADP to P2Y_12_ stimulates activation of GPIIb/IIIa receptor resulting in enhanced platelet degranulation and thromboxane production, consequently, prolonged platelet aggregation. The first of this class was ticlopidine, but despite the promising efficacy, its use was limited by several side effects, including neutropenia and thrombocytopenic purpura. Development of clopidogrel followed and showed an improved safety profile compared to ticlopidine. This led to dual platelet antiaggregant therapy with aspirin and clopidogrel becoming standard therapy for many treatments [109,110,111].

Glycoprotein IIb/IIa receptor antagonists, abcximab and tirofiban, represent another strategy in the inhibition of fibrinogen-mediated platelet activation and treatment for PCI and ACS. Research interest in these antagonists has been dimmed due to negative data, including mortality and significant increase in bleeding [109,110,111]. Despite these findings, tirofiban shows short half-life, leading to beneficial effects when used in the perioperative context of cardiac surgery. Several studies showed reduced myocardial infarction, decreased hemorrhage and transfusion requirements in ACS patients undergoing urgent on-pump coronary bypass grafting surgery. Moreover, administration of tirofiban is an established strategy for treatment of heparin-induced thrombocytopenia [111,112,113,114,115].

The selective inhibitors of COX-2, an enzyme directly involved in inflammatory processes that are routinely used to treat arthritis because the absence of gastric toxicity are not acceptable substitutes to aspirin^®^ in patients requiring platelet antiaggregant therapy for cardiac protection [103]. These inhibitors do not act on the production of TXA_2_ and present significant risks due to cardiovascular side effects on the endothelium [116].

By limiting the ability of platelets to aggregate, the platelet antiaggregant drugs help in preventing the formation of thrombi that can block blood vessels and lead to acute myocardial infarction or cerebrovascular accident (CVA). In high-risk patients, aspirin^®^ reduces the risk of first acute myocardial infarction by 20%, which may reduce the risk of recurrence in some 30% of patients. Similarly, platelet antiaggregant agents may reduce the risk of stroke or recurrent ischemic accident transition, also preventing the occlusion of vessels that were previously removed with stent. New clinical and experimental evidences suggest that platelet antiaggregant agents, neglected in the treatment of acute pulmonary embolism, may prevent the initiation and propagation of venous thrombi, minimizing the adverse physiological consequences of acute pulmonary embolism [81].

The resistance to aspirin^®^ has been described in the literature and can be defined as an inefficiency of aspirin^®^ in inhibiting the production of TXA_2_ and/or TXA_2_-dependent platelet function (e.g., platelet aggregation), or as the inefficiency of aspirin^®^ in preventing ischemic events in atherothromboembolic infarct patients and users of aspirin^®^ [66,92,102,111,116,117]. Although aspirin^®^ reduces arterial thrombosis (10%–20%), some patients still suffer at least one recurrent arterial thrombotic event during long-term treatment [103,118,119]. This resistance is significantly associated with increased risk of myocardial infarction, stroke, and death in comparison of resistant patients with those sensitive to aspirin^®^ (24% *vs.* 10%) [120].

The resistance to aspirin^®^ may be due to several factors, including: (a) Variation in the bioavailability of aspirin^®^; (b) Platelet dysfunction; (c) Polymorphisms; (d) Platelet interaction with other blood cells or molecules; (e) Smoking; (f) Excess adrenaline due to physical or mental stress; (g) Biosynthesis of PGF2α; (h) Increased sensitivity to collagen; (i) Interference of other NSAIDs, seen in some patients who have higher levels of urinary thromboxane, although subject to high doses of aspirin^®^ [120,121,122]. The process of resistance is not exclusive to aspirin^®^, but also described for clopidogrel and inhibitors of α_IIb_β_3_ [66,97,123,124].

Similar to anticoagulants therapy, platelet antiaggregant therapy requires monitoring of blood tests, mainly due to high incidence of non-responsiveness to aspirin^®^/clopidogrel, and resistance to inhibitors of α_IIb_β_3_. The resistance pattern reaches up to 25% of the percutaneous transcardiac intraluminal angioplasty and contributes to the outcome of these stents [43]. Currently, several studies work on developing and testing kits for easier access to the platelet function and response to individual platelet antiaggregant agents. Thus, the choice of a treatment with greater efficiency and less side effect for a given patient can be performed with greater precision [79,118,120,121,125,126].

## 9. Conclusions

Besides being enucleated, platelets are essential for primary haemostasis. Platelet disorders comprise a large and heterogeneous group of bleeding diseases that range in severity from mild to severe. Thus, the platelet, their receptors and enzymes become important therapeutical targets for designing new drugs by treatment of high incidence pathological processes. In conclusion, the comprehension of platelets’ structure and intra-platelet function might be useful for further studies on the role of platelets in the important haemostatic process.
